# Desert locust (*Schistocerca gregaria*) flour as an emerging functional ingredient for baking flavorful and nutritious whole wheat bread

**DOI:** 10.1016/j.afres.2025.100802

**Published:** 2025-06

**Authors:** Chrysantus M. Tanga, Antonny M. Nzomo, Paul N. Ndegwa, Sunday Ekesi, Fathiya M. Khamis, Komivi S. Akutse, George Ong'amo, Brian O. Ochieng, Margaret Kababu, Dennis Beesigamukama, Shaphan Y. Chia, J․Ghemoh Changeh, Sevgan Subramanian, Thomas Dubois, Segenet Kelemu

**Affiliations:** a International Centre of Insect Physiology and Ecology, P.O. Box 30772–00100, Nairobi, Kenya; b Department of Zoology, University of Nairobi, P.O. Box 30197-00100, Nairobi, Kenya; c International Centre for Tropical Agriculture (CIAT), P.O. Box 823 – 00621, Nairobi, Kenya; d Unit for Environmental Sciences and Management, North-West University, Potchefstroom, 2520, South Africa

**Keywords:** Locust rearing, Processed locust powder, Functional ingredients, Wheat bread fortification, Aroma compounds, Food security

## Abstract

Increasing interest in consuming insects is accelerating the expansion of the fortified snacks market. However, there is limited knowledge of the drivers of consumers to appreciate the role of desert locust [DL] *Schistocerca gregaria* Forskål as emerging functional ingredient for use in the food industry. Here, we evaluated the nutrient profile and functional properties of wheat breads fortified with powder from captive mass reared DL. The crude protein values of processed DL meal fed on three different food plants ranged between 40 and 56 %. The specific volume and firmness of the breads correlated positively and negatively, respectively, with incremental levels of DL flour. The brightness of the breads’ crumb and crust decreased, while the colour differences and the redness progressively increased with DL flour inclusion levels. Protein, fat, ash and amino acid content were significantly higher in bread fortified with DL flour. Calcium, iron and zinc levels were 2.0–2.5, 1.1–1.3 and 1.1–1.3-fold higher, respectively, in breads fortified with DL. The most pleasant aroma compounds were pronounced in the breads enriched with DL. This work demonstrated that DL meal can be integrated into wholesome yeast-leavened products to strengthen the growing consciousness of end-users towards innovative and sustainable use of these products in food relief programs to overcome malnutrition.

## Highlights

• Desert locust powder [DLP] is a novel baking ingredient with healthier protein (42–56 %).
• Inclusion of 10 % DLP to bread recipe increased protein, amino acids, calcium, iron and zinc in bread.
• Breads enriched with desert locust flour had the most pleasant aroma compounds.
• Bread with DLP can be assigned as promising solution to the impending food crisis.

## 1 Introduction

*Schistocerca gregaria* (i.e., desert locust) is one of the most dominant edible insect species, widely spread across Africa, Australia, Asia, and Europe (Peng et al., 2020). Desert locust is highly voracious, can migrate over long distances and has a high speed of reproduction which renders it one of the most serious agricultural pest across the globe (Kassegn & Endris, 2021; Moharana et al., 2020). A swarm of desert locust can contain up to 10 billion insects that consumes leaves, shoots, flowers, bark and fruits of every green plant they encounter on their way.

Desert locust have previously been utilized as a source of food since the antique times (van Huis, 2013). Evidence suggests that locusts have been widely collected and consumed as food during outbreaks across Africa and Asia (Egonyu et al., 2021). They can be consumed raw after removing wings and legs, or prepared by frying, toasting, boiling and sun-drying or only toasting prior to consumption (Gahukar, 2020; van Huis et al., 2003). Desert locusts can be harnessed as a novel alternative protein for food and feed (Egonyu et al., 2021; Paul et al., 2016; Siddiqui et al., 2023). They are rich in proteins (52–76 %), fats (12–32 %), carbohydrates (1–19 %), vitamins, fatty acids, minerals and amino acids, critical for human health and nutrition (Das & Mandal, 2013; Kekeunou et al., 2020; Zielińska et al., 2015). They have appreciable levels of flavonoids and can convert phytosterols obtained from plants into useful derivatives utilizable as food ingredient or in formulation of high value products like pharmaceuticals (Cheseto et al., 2015, 2020; Kinyuru, 2020).

However, the desert locust swarms can reportedly destroy up to 20 percent of global's cropland, ruining the livelihoods of millions of people (Worku et al., 2022) thereby potentially jeopardizing food security (Kassegn & Endris, 2021; Kimathi et al., 2020). Therefore, in the contemporary society, the need for sufficient food production that is commensurate with the dietary requirements of the exponentially rising world population has necessitated chemical repression of the locust. Hence, wild collection of desert locust for human consumption may pose serious chemical hazards that may be detrimental to human health. There is need for regulated cage rearing to produce locust with meagre residual chemicals for human consumption.

Insect rearing has been practiced for 7000 years (Rumpold & Schlüter, 2013) however, to date, over ninety-two [92 %] percent of insect species are still being collected in the wilderness (Yen 2015a). But wild collection has been associated with safety issues and extinction of species. Mass rearing could ensure stable supply of insect products and cushion against seasonal shortages sustaining the market needs (Raheem et al., 2019). The rearing strategies optimizes factors such as temperature, humidity, light/illumination, rearing container properties, ventilation, oviposition site, larvae/population density, food composition, food and water availability, food quality as well as microbiological contamination to guarantee optimal yields (Rumpold & Schlüter, 2013). Previous studies have indicated that combination of the food crops: *Phaseolus vulgaris* and *Zea mays* or *P. vulgaris* enhanced propagation of locust recommending them for extensive and small-scale locust rearing intended for commercial utilization (Mphephu, 2023). Even as chemical exposure to edible insects may be reduced through in-house rearing of locusts, allergenicity has been another risk factor associated with edible insects’ and are reportedly thermal potent and resistant digestive enzymes (Ribeiro et al., 2021, 2018). Reports have indicated that most individuals expressing allergenicity to insects are subjects allergic to crustaceans or with long exposure to insects (Ribeiro et al., 2021). However, despite sensitivities among human subjects to locust being reported (Pener, 2014; Ribeiro et al., 2018), allergenicity cases against edible insects are generally low but are pronounced only in individuals with pre-existing allergic reactivities.

Irrespective of wild collection or mass rearing, the consumption of locusts, like other edible insects, generally remains low due to neophobia and low acceptability of whole edible insects (Elhassan et al., 2019; Gravel & Doyen, 2020; Olum et al., 2020). Thus, to increase consumer acceptability and counter aversion to entomophagy, insects have been milled and incorporated into formulations of functional foods (Melgar-Lalanne et al., 2019). Recently, the desert locusts have been widely used in the formulation of processed products like flavored snacks, baked products and energy bars, among others, with high consumer acceptability (Cheseto et al., 2020; Haber et al., 2019; Mishyna et al., 2019). Processed products fortified with locusts have good sensory characteristic and are rich in proteins, minerals, fibre, sterols and flavonoids, and can therefore be used to mitigate malnutrition and improve health outcomes, especial among children and women of childbearing age (Althwab et al., 2021; Cheseto et al., 2020; Haber et al., 2019). This study therefore physiochemically examined breads fortified with flours from captive mass reared desert locust fed on food security crops that are widely damaged during outbreaks to promote the sustainable and safe use of desert locust powder as novel functional ingredients.

Yeast-leavened bread is extensively considered a major staple food in numerous countries worldwide, especially consumed daily at household levels (Arranz-Otaegui et al., 2018). In many countries, bread is being considered a cheap and complete basic auxiliary food that can be utilized in extreme poverty (Kourkouta et al., 2017). However, bread is made from nutritionally imbalanced wheat as the main ingredient, and therefore revamping its nutritional profiles with novel ingredients has been advocated (Agu et al., 2010; Lu et al., 2018). Accordingly, authors have successfully blended wheat flour with pseudocereals such as quinoa, amaranth and buckwheat purposefully to enhance the nutritional quality, bolster the anti-oxidative and functional properties and dilute the gluten contents of the resultant bakery products (Alvarez-Jubete et al., 2010; Demin et al., 2013; Giménez-Bastida & Zieliński, 2015; Tosi et al., 2001). Likewise, Several edible insects, including locusts, have been used in food fortification with diverse outcomes on their sensory features, increased digestibility, improved macro and micro nutrient content as well as consumer acceptability in comparison to ordinary bread products (Althwab et al., 2021; Cappelli et al., 2020; da Rosa Machado & Thys, 2019; Gantner et al., 2022; Haber et al., 2019; Mihaly Cozmuta et al., 2022; Ochieng et al., 2023b). On the contrary, integration of insect ingredients in doughs may compromise the shelf-life of the developed products due to high fat content which enhance susceptibility of the products to oxidative rancidity and may modify the functional properties of baked goods that may facilitate moisture retention. Regardless, due to its widespread consumption, fortification of bread with high protein ingredients such as insect meal can serve as a vehicle to curb widespread malnutrition and food insecurity. For instance, Althwab et al. (2021) realized a decrease in specific volume and an increase in protein, essential amino acids (except lysine) and sensory acceptability of bread with increase in migratory locust flour incorporation levels. Integrating *A. domesticus, H. illucens* and *T. molitor* into wheat flour enhanced the protein and fat contents of resultant breads (González et at., 2019). Finally, a review composed by Amoah et al. (2023) discovered that enrichment of bakery products including bread with insect flours directly enhance the nutritional components of products.

## 2 Methodology

### 2.1 Ethical permits

According to strict adherence to all the provisions provided by the Institutional Animal Care and Use Committee (IACUC) of Kenya Agricultural and Livestock Research Organization (KALRO)-Veterinary Science Research Institute (VSRI) [KALRO-VSRI/IACUC028/16032022], Muguga North, this research work was approved for implementation. Furthermore, this study was assessed and ratified by the National Council for Science Technology and Innovation in Kenya (NACOSTI/P/21/8303) and the University of Nairobi, Nairobi, Kenya, which provides permission to undertake plant sample collection from the field, processing, identification and experimental research in the laboratory.

### 2.2 Survey of food crops devastated by locust outbreak

**Fig. 1:**
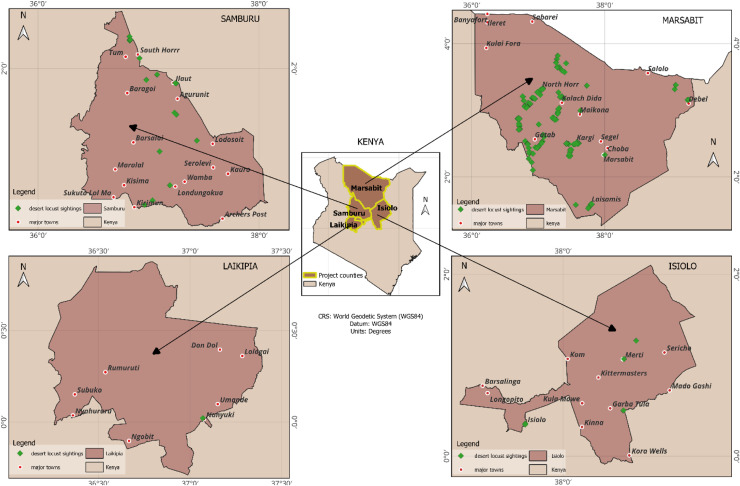
A map of areas highly affected with massive crop during the devastating desert locust outbreaks in Northern Kenya.

Field surveys were conducted in farms across Marsabit, Samburu, Isiolo and Laikipia Counties in Northern Kenya to identify crops that were heavily infested desert locusts. Destructive sampling technique was used where approximately five infested leaves were randomly plucked per plant or the entire above ground parts harvested if the plant was small in size. Plant portions from each farm were processed, packed separately in perforated paper bags, and ferried to the laboratory in cooler boxes for identification. Global positioning system (GPS) data was collected for all the farms visited and mapped to illustrate the range of host plants distribution (Fig. 1). Photos of the fresh plant specimens were taken, after which the plants were dried using a plant press. Identification of the plants was done at the Kenya Forest Research Institute (KEFRI) and confirmed by the Kenya Plant Health Inspectorate Service (KEPHIS) Plant Quarantine Laboratory and the National Museums of Kenya (NMK) using the identification keys of Kenya trees and shrubs. The plant nomenclature used was in line with the International Plant Names Index database and Missouri Botanical Garden database. Voucher samples were deposited at the Biosystematics Unit of *icipe*, NMK and KEFRI.

### 2.3 Desert locust stock culture

Desert locusts in a gregarious phase were acquired from the mother stock colony at the Animal Rearing and Containment Unit (ARCU) at the International Centre of Insect Physiology and Ecology (*icipe*), Nairobi, Kenya. The *icipe* colony was established using juveniles and adults obtained from Desert Locust Control Organization for Eastern Africa [DLCO-EA], Nairobi, Kenya. *icipe*’s colony is mass-reared on wheat (*Triticum aestivum*) seedlings and periodically infused with field samples to maintain genetic vigor, prevent inbreeding depression, and disease transfer.

Two hundred desert locusts were reared in aluminum cages measuring 50 × 50 × 50 cm, fitted in a well-ventilated room of size 4.5 × 4.5 m at a temperature of 30 ± 4 °C, 40–50 % relative humidity (RH) and a photoperiod of 12 h light (L) and 12 h darkness (D). Incandescent light bulbs (40 W) were fitted on the upper surface of all the experimental cages to provide heat. The locusts were fed on young seedlings of wheat and barley supplemented with wheat bran and water provided in cotton balls. Long tubes (3.5 cm wide × 9 cm) with 70 % sterilized silica sand were placed at the bottom of the cages for egg-laying. The eggs were incubated at 32 ± 1 °C and newly emerged nymphs were introduced into aluminum cages and fed with wheat bran and fresh wheat seedling leaves ad libitum. Moistened cotton wool rolled into balls (70 %) were then transferred into the cages to regularly supply water and was replaced after every 2 days. The cages were monitored daily, dead locusts were removed and mortalities recorded. The experimental colony was grown for at least five generations prior to the commencement of the experiment on selected food crop identified to be the most affected during locust outbreaks in Section 2.2.

### 2.4 Food plants and rearing conditions for desert locusts

Newly emerged neonates (∼ 24 h old) were introduced into standard metallic rearing cages (30 cm × 30 cm × 30 cm) comprising of translucent Perspex front with 3 opaque sides and kept at low populations to avoid overcrowding and stressful surrounding (Sørensen & Loeschcke, 2001). The bottom of the cages was built of polyvinyl chloride with stainless steel metal wire mesh on the top (16 × 16 cm) to allow for adequate ventilation. At the back of the cage, an opening was made and a round sleeve fixed to allow access for the transfer of plant materials and locusts. The neonates were fed on seedlings of three major desert locust host plants identified: common beans (*Phaseolus vulgaris* Linnaeus), sorghum (*Sorghum bicolor* Linnaeus) and wheat (*Triticum aestivum* Linnaeus). Fresh host plant leaves (15–20 cm length) and 70 % moist cotton balls were placed in the cages. The cages were cleaned daily by removing remains of previous food plant, insect waste and dead insects.

The plant seedlings were grown and maintained in the screenhouse in 2 litre plastic containers each of diameter 17 cm filled with soil, compost and moist sand at the ratio of 3:2:1 (v/v/v), and the set up was watered daily (Campbell & Nes, 1983; Ochieng-Odero et al., 1994). One hundred and fifty seeds of each host plant were planted and raised without chemical pest management. The screenhouse conditions were maintained at room temperature [25±1 °C], relative humidity [65±5 %] and a photoperiod of 12h:12 h for light and dark to promote optimal growth and development.

### 2.5 Nutrient profiling of host plant and *Schistocerca gregaria* powder

A kg of each food plant was harvested (without the roots) and chopped into small pieces (5 mm) and placed on 3050 mm × 2030 mm sized clean transparent plastic sheets with circulating dry air at an ambient temperature of 28 ± 2 °C for 48 h with a wall fan heater. The samples were milled in a grinder (Retsch GM200, Haan, Germany) fitted with 0.4 mm screen. The ground samples were labeled, and individually stored in airtight but well-sterilized transparent glass bottle containers at room temperature for further analysis. Different life stages of desert locust (5th instar nymphs, mated adults and ovipositing adults) (700 each) fed on the various food plants were dried using a drying machine (SDO-225, Wagtech International, Thatcham, UK) at exactly 60 °C for 48 h. The dried desert locusts were further processed into powder in a mechanical grinder.

The dry matter of the different samples were analyzed using the oven drying method at 100 °C for 48 h (AOAC, 1990). The minerals, sugar, starch, digestibility and oil contents of the dried food plants were determined using a near infrared spectrometer (Thermo Fisher Scientific, Verona, USA). The proximate composition of dried food plants and selected life stages of the locust were established. Crude protein contents were estimated using the Kjeldahl method (AOAC, 1990). Constant factors of 4.25 (Finke, 2007) for the food plants and 5.33 (Boulos et al., 2020) for the locust were considered for nitrogen to protein conversion. The ash contents were established by sample kindling in a muffle oven whereby the samples were exposed to higher temperatures of about 550 °C for a period of 3 h (AOAC, 1990). A fibre analyzer (FIWE 6, VELP Scientifica, Usmate Velate, Italy) was used to determine the neutral detergent fibre (NDF) and acid detergent fibre (ADF) following the procedures outlined by van Soest et al. (1991). The Velp solvent extractor (Velp SER 148, Velp Scientifica, Europe) was readily used to established the fat content with ethyl ether as an extractant (AOAC, 1990). The gross energy content (GE; MJ/kg) was determine indicated: [(17.6)+(0.0617Protein)+(0.2193Fat)+(0.0387Fibre)]−0.1867Ash (Sauvant et al., 2004). Each analysis was replicated three times.

### 2.6 Bread formulation and baking

**Fig. 2:**
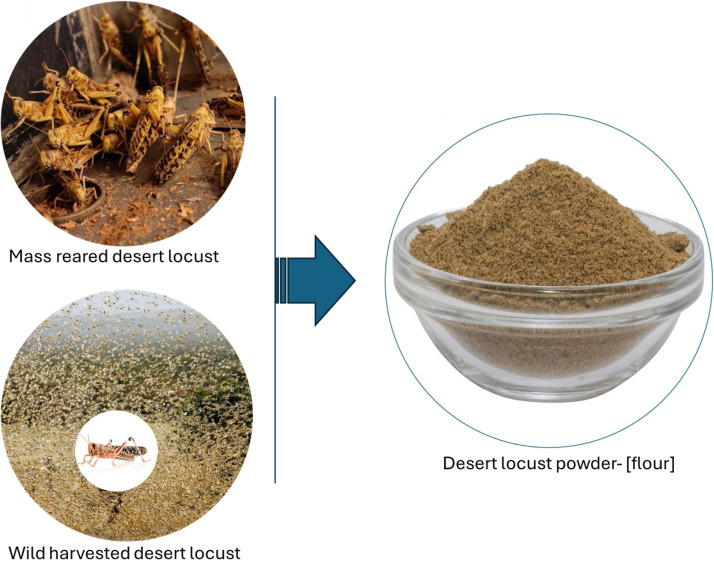
Mass reared desert locusts or wild harvested desert locusts can be processed into high-quality powder or flour as novel baking protein-rich ingredient.

For the baking, the following ingredients [all-purpose wheat flour (Exe, Unga Limited, Nairobi, Kenya), yeast, sugar, salts and shortening] were bought from a nearby market in Nairobi, Kenya. Flours from adult desert locust reared on *Phaseolus vulgaris* was considered for bread development due to the higher protein content expressed (Table 3). Adult locusts were preferred due to their reportedly high fat and protein content (van Huis et al., 2013), their ease of mass collection (DeFoliart, 1999), their palatability and digestibility (Banjo et al., 2006; Mitsuhashi, 2016) and economic benefits of their large edible biomass (Ayieko et al., 2010). The locusts (2 kg) were fasted for 24 h, freeze-killed at −80 °C for 2 h and blanched in hot water for 3 min. The locusts were then dried and processed into powder [Fig. 2]. Macro-nutrients of the wheat flour and the locust flour were characterized according to procedures described in Section 2.7 (Table 1). Flour blends were composited by mixing wheat flour with locust powder at ratios of 100:0 (control), 95:5 and 90:10 (w/w).

**Table 1:** Proximate analyses (g/100g dry matter basis) of *Schistocerca gregaria* and wheat flour used for bread development.

Source of flour	Proximate parameters					
	Dry matter	Moisture	Protein	Fat	Ash	Fibre
*S. gregaria*	89.37 ± 0.66	10.63 ± 0.66	44.93 ± 1.73	17.53 ± 2.16	8.30 ± 1.46	4.87 ± 0.12
Wheat	87.90 ± 0.00	12.10 ± 0.00	12.60 ± 0.06	1.74 ± 0.06	2.50 ± 0.02	0.59 ± 0.02
t-value	2.23	−2.23	23.1	7.31	3.98	35.06
*df*	4	4	4	4	4	4
*p*-value	0.089	0.089	2.08E-05	0.002	0.016	3.95E-06

Methods described by de Oliveira et al. (2017) was adopted for preparing the bread products. The dry ingredients, included wheat powder (as control), wheat-locust flour blends (300 g), yeast (9 g), sugar (15 g) and salt (6 g), were mixed in a kitchen mixer (Kenwood, Havant, UK) on a low speed for 6 min. The other ingredients – vegetable fat (6 g), and water – were then added and further blended for exactly 10 min till a consistent dough was fully developed. The dough was then weighed, cut into sizeable pieces, moulded and placed in greased baking pans. The doughs were proofed for 40 min at 85 % RH and subsequently allowed to ferment at 37 °C and close to 90 % RH for over 30 min. Fermented doughs were then cooked in an oven cooker (Bistrot 665, BestFor®, Ferrara, Italy) at 200 °C for 20 min. The process was replicated thrice with different dough batches for every wheat substitution level to yield breads.

### 2.7 Physical properties of the breads

#### 2.7.1 Specific volume

The volume of fresh breads were examine using the methods described by Datta et al. (2007). Volume values from triplicate determinations were expressed as specific volume and calculated as volume displaced by seed/weight of the product.

#### 2.7.2 Texture of bread products

The texture profile analysis (TPA) was adopted for the determination of bread firmness according to AACC (2000) using a texture analyzer (Stable Micro Systems, Godalming, UK; Software: Exponent Version 6,1,16,0). A reversible blade HDP/BS probe was used (36 mm in diameter). The measurements were accomplished with the following settings: compression test mode, 1.00 mms^-1^ pre-test speed, 2.00 test speed, 10 mms^-1^ post-test speed, 40 % strain for the target mode and automatic trigger of 5 g force. The measurements were taken in triplicate.

#### 2.7.3 Colour of bread products

A spectrophotometer (Konica Minolta Sensing, Osaka, Japan) (Kowalski et al., 2022) was used to determine the colours of bread crumb and crust. The design of experiment was made to follow the colour system in the L* a* b* (or CIE L*a*b*) space to evaluate the L* values (brightness) and a* and b* (chromaticity coordinates). Mean colour parameters were key in calculating the total colour difference according to Equation 1;

(1) $$\Delta E=\sqrt{{\left(\Delta L^{*}\;\right)}^{2}+{\left(\Delta a^{*}\right)}^{2}+{\left(\Delta b^{*}\right)}^{2}}$$

where:

Δ*a* = redness difference; Δ*L* = brightness difference; Δ*b* = yellowness difference.

### 2.8 Proximate determinations of the bread

Proximate parameters were assessed on wheat flour, locust flour and formulated bread samples according to AOAC (AOAC, 2012). Oven-drying for 3 h at 130 °C was applied to record the moisture levels in the bread. Kjeldahl method for nitrogen content evaluation and a nitrogen to protein conversion factor of 5.33 was adopted for protein content computation as recommended by Boulos et al. (2020). The samples were put in a muffle furnace with a temperature of 550 °C for 3 h to get the ash content. The approach reported by van Soest et al. (1991) was used to analyse for the neutral detergent fibre (NDF) and acid detergent fibre (ADF), whereas crude fibre was gravimetrically determined after 0.25 % sulphuric acid and 0.25 % sodium hydroxide digestion in a Velp fibre analyzer. Velp solvent extractor was critical for determining the fat content of the samples with ethyl ether being considered as the extractant. Carbohydrate contents were determined as difference between 100 and other macromolecular proximate components. Gross energy was calculated by the At Waters method according to Food and Agriculture Organization of the United Nations (FAO) (FAO, 2003). The analysis of each treatment was replicated three times.

### 2.9 Amino acid profile of the bread products

Bread products (100 mg) were hydrolyzed using 6 mol/L HCl under nitrogen atmosphere at a temperature of 110 °C for a period of 24 h. The hydrolysates were then dried under vacuum atmosphere before reconstituting in 1 millilitre mixture of acetonitrile/0.01 % formic acid(5:95). The mixture was subjected to 30 s of vortexing and 30 min of sonication, and then centrifuged for 15 min at 14,000 rpm. Filtered supernatants using 0.45 μm syringe filter were analyzed accordingly using the liquid chromatography mass spectrometry (LCMS) (Agilent Single Quadrupole LC-MS 1200 series, Agilent, Massachusetts, USA). The separations through the chromatography were achieved using a Zorbax RX-C18, 4.6 × 250 mm, 5 μm column, operated at 40 °C. The gradient elution program, instrument operating conditions and quantification of the individual amino acids followed the protocols outlined by Cheseto et al. (2017). All the analyses were performed in triplicates.

### 2.10 Mineral content of bread

Ground bread products (0.5 g) were dampened with 2 mL of 9.8 mol/L hydrogen peroxide (Sigma- Aldrich) and 8 mL of 16.2 mol/L nitric acid (VWR Chemicals, Fontenay- sous-Bois, France) and allowed to wet ash over-night. The bread types were later exposed to well calculated programmed temperature digestion (75 °C/30 min, 120 °C/20 min, 180 °C/20 min and 200 °C/10 min). Mineral content of various digests were further determined in consonance with the method reported by Ochieng et al. (2023a) using inductively coupled plasma optical emission spectrometry (ICP-OES) (Optima 2100 ™DV ICP-OES, Perkin Elmer, MA, USA) and calibration curves of mineral standards.

### 2.11 Volatiles of organic compounds (VOCs) from various bread products

The volatile profiles were identified and quantified following approaches previously described and well documented in literature (Cheseto et al., 2020; Mekonnen et al., 2021). Briefly, headspace volatiles from bread samples (10 g) were trapped on precleaned Super-Q traps (30 mg) (Analytical Research System, Gainesville, FL, USA) for 24 h. The system comprised of a push-pull Gast pump (Gast Manufacturing, Benton Harbor, MI, USA) and Vacuubrand CVC2 vacuum pump (Vacuubrand, Wertheim, Germany), which were often used to introduce activated charcoal-filtered and moisturized air on the samples at a flow rate of 340 mL/min through the Super-Q traps. Volatiles adhered on the Super-Q traps were eluted with 200 µL of dichloromethane (Merck, Darmstadt, Germany) into 250 µL glass inserts (Supelco, Bellefonte, PA, USA) fitted into 2 mL glass vials and immediately analyzed by a GC–MS on an HP 7890A series gas chromatograph (Agilent Technologies, Wilmington, NC, USA) attached to an HP 5975C mass spectrometer (Agilent Technologies). All experiments were replicated three times for each treatment.

### 2.12 Data analysis

R software version 4.3.1 (Posit team, 2023) was used to undertake all the data analyses in this study. The normal data sets distribution and homoscedasticity of variances was tested using Shapiro-Wilk and Bartlett tests, respectively. Significant differences among means of data groups of test parameters were analyzed using ANOVA. ANOVA with Welch's F-test was used in cases where the normality and homoscedasticity test were violated. Mean values for the various treatments were contrasted by the Tukey test, with 5 % level of significance. A multidimensional relationship among nutritional, physical properties and locust inclusion levels of bread were assessed using Principal Component Analysis (PCA). To analyse the chemical profiles of the breads formulated with varying locust flour levels, one-way analysis of similarities (ANOSIM) utilizing the Bray–Curtis dissimilarity matrix was used. The impact of the different volatile compounds to the variation between volatiles from different breads were computed and visualized using the non-metric multidimensional scaling approach based on the similarity percentages (SIMPER) analysis.

## 3 Results

### 3.1 Nutritional composition of the host plants

The nutritional contents of the three experimental host plants are presented in Table 2. *Phaseolus vulgaris* expressed higher protein content, digestibility, energy, sugar and iron than *Triticum aestivum* and *Sorghum bicolor*.

**Table 2:** Nutritional composition (on dry matter basis) of the food plants for rearing Schistocerca gregaria Forskal.

Parameter	Experimental food plants		
	*Phaseolus vulgaris*	*Triticum aestivum*	*Sorghum bicolor*
Boron (mg/100g)	5.24	1.35	3.75
Molybdenum (mg/100g)	<0.01	0.20	0.22
Iron (mg/100g)	128.00	14.50	87.90
Copper (mg/100g)	1.18	0.83	1.05
Zinc (mg/100g)	2.49	5.51	2.26
Manganese (mg/100g)	6.53	9.28	10.30
Sodium (mg/100g)	12.40	213.00	14.70
Sulphur (g/100g)	0.19	0.22	0.16
Magnesium (g/100g)	0.54	0.22	0.26
Potassium (g/100g)	2.34	1.97	1.70
Phosphorus (g/100g)	0.34	0.93	0.26
Calcium (g/100g)	3.40	0.15	0.45
Sugar (g/100g)	12.7	9.17	1.42
Starch (g/100g)	<0.10	<0.10	<0.10
Protein (g/100g)	31.9	24.9	17.9
Fibre (g/100g)	9.00	23.1	27.7
Ash (g/100g)	14.3	6.65	10.2
Acid detergent fiber (g/100g)	13.2	38.1	47.0
Neutral detergent fiber (g/100g)	17.6	40.7	62.2
Digestibility (NCGD) (g/100g)	80.8	63.2	57.5
Oil (g/100g)	6.49	6.52	2.46
Energy (MJ/kg)	13.2	10.7	8.70
Dry matter (DM) (g/100g)	86.1	90.4	89.5

NCGD: Neutral cellulase gammanase digestibility.

### 3.2 Effects of food plant diets on the proximate composition of successive life stages of *Schistocerca gregaria*

The proximate composition of the various successive life stages of *S. gregaria* fed on various host plant diets are presented in Table 3 and varied significantly across the different food plant diets. Mating adults fed on *T. aestivum* recorded the highest crude fat content (22 %), while ovipositing females fed *P. vulgaris* had the lowest crude fat values (4 %). The crude protein level of ovipositing females fed *P. vulgaris* was significantly higher compared to the other food plant diets. Protein values recorded for the different nymphal stages when fed on the different food plant diets varied considerably (Table 3). The protein values of the 5th instars (F_(2,3__)_ = 42.24, *p* = 0.0064) and ovipositing female (F_(2,3__)_ = 25.32, *p* = 0.0050) fed on *P. vulgaris* were significantly higher compared to the other diets, while that of mated adults were similar across the various food plant based diets.

**Table 3:** Proximate composition (% DM) (± SE) of the various life stage of *Schistocerca gregaria* Forskal fed on different food crops.

Life stage	Food crops	Dry matter	Ash	Crude protein	Crude fat	ADF	NDF
5th nymphal instar	*Phaseolus vulgaris*	15.0 ± 0.0^a^	7.9 ± 1.5^a^	46.34 ± 0.3^a^	13.7 ± 2.2^a^	7.3 ± 0.4^b^	11.61 ± 1.29^a^
	*Triticum aestivum*	12.0 ± 0.5^a^	10.7 ± 1.1^a^	41.74 ± 0.4^b^	13.6 ± 1.4^a^	9.1 ± 0.5^b^	12.94 ± 0.83^a^
	*Sorghum bicolor*	7.5 ± 0.3^b^	10.8 ± 3.8^a^	40.31 ± 1.2^b^	15.2 ± 0.0^a^	12.0 ± 0.0^a^	19.43 ± 1.86^a^

Mated adults	*Phaseolus vulgaris*	89.7 ± 0.3^a^	10.4 ± 2.4^a^	47.51 ± 2.5^a^	14.8 ± 0.2^b^	11.7 ± 0.0^b^	21.5 ± 0.3^b^
	*Triticum aestivum*	88.1 ± 0.0^b^	9.0 ± 2.3^a^	42.38 ± 0.1^a^	21.8 ± 0.7^a^	13.7 ± 0.4a^b^	23.3 ± 0.4^b^
	*Sorghum bicolor*	90.3 ± 0.3^a^	5.5 ± 1.1^a^	44.92 ± 0.5^a^	16.0 ± 0.2^b^	16.3 ± 1.0^a^	30.7 ± 1.0^a^

Ovipositing females	*Phaseolus vulgaris*	88.5 ± 0.0^b^	5.6 ± 0.6^a^	55.81 ± 0.0^a^	3.9 ± 0.43^c^	20.8 ± 0.5^a^	38.32 ± 0.24^a^
	*Triticum aestivum*	90.3 ± 0.2^a^	5.8 ± 0.6^a^	52.37 ± 2.6^b^	5.9 ± 0.0^b^	17.5 ± 1.2^ab^	39.72 ± 0.80^a^
	*Sorghum bicolor*	90.0 ± 0.0^a^	6.1 ± 0.5^a^	45.69 ± 0.6^b^	16.8 ± 0.0^a^	14.0 ± 0.4^b^	26.99 ± 0.01^b^

Mean values in each column with the same letters were considered not to be significantly different at 5 % level of probability.

### 3.3 Bread physical characteristics

**Fig. 3:**
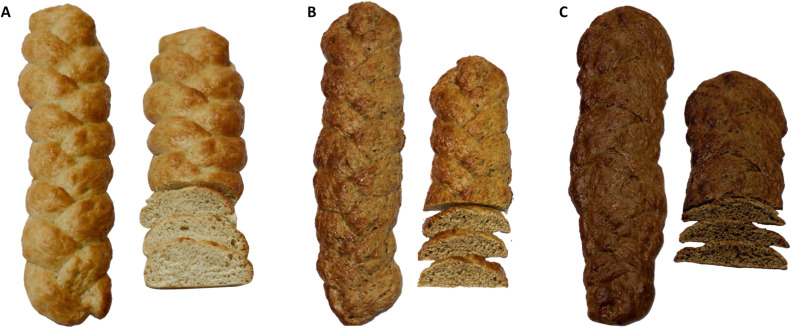
Appearances of the different bread types: control bread (A), breads enriched with 5 % locust flour (B) and bread enriched with 10 % locust flours (C).

The physical characteristics assessed showed significantly variation (*p* < 0.05) among the different bread types (Table 4). Control breads depicted significantly (*p* < 0.05) higher specific volume which progressively reduced with increasing locust flour blends (Fig. 3) whereas firmness significantly increased with the rising substitution levels. L* of both crust and crumb significantly decreased with the rising levels of locust flour inclusion (Fig. 3). Conversely, the a* in both the crust and crumb increased with the increasing levels of locust flour integration into bread. The ∆*E* in both the crust and crumb correlated with the wheat substitution level.

**Table 4:** Physical traits of breads integrated with different inclusion levels of desert locust and wheat flours.

Parameter	Locust flour levels			F	DF	P value
	0 %	5 %	10 %			
Specific volume (cm3/g)	4.48 ± 0.06^c^	4.05 ± 0.02^b^	3.59 ± 0.01^a^	167.48	2,6	0.001
Firmness (g.sec)	1844.00 ± 45.80^a^	3500.61 ± 238.00^b^	6354.48 ± 180.35^c^	171.11	2,6	0.001
Crust colour						
L*	59.57 ± 0.16^c^	49.91 ± 0.11^b^	39.99 ± 0.33^a^	2006.5	2,6	0.001
a*	8.27 ± 0.08^a^	10.04 ± 0.11^b^	13.49 ± 0.30^c^	191.73	2,6	0.001
b*	24.51 ± 0.01^c^	23.00 ± 0.28^b^	21.60 ± 0.09^a^	74.39	2,6	0.001
∆*E*	–	9.93 ± 0.29^a^	20.46 ± 0.28^b^	683.46	1,4	0.001
Crumb colour						
L*	68.20 ± 0.58^c^	52.90 ± 0.02^b^	42.41 ± 0.92^a^	425.85	2,6	0.001
a*	3.15 ± 0.01^a^	5.63 ± 0.00^b^	7.56 ± 0.02^c^	32,433	2,6	0.001
b*	16.80 ± 0.04^a^	18.61 ± 0.01^b^	19.13 ± 0.10^c^	400.8	2,6	0.001
∆*E*	–	15.61 ± 0.56^a^	26.28 ± 0.78^b^	123.41	1,4	0.001

Values are shown as mean ± SE. Values in each rows denoted by various letters varied significantly (*p* < 0.05). L*: brightness; a*: redness; b*: yellowness.

### 3.4 Proximate composition and energy content of bread formulated with locust flour

The levels of protein, moisture, fibre, dry matter, fat, carbohydrates, ash and gross energy contents significantly (*p* < 0.05) varied across the locust flour inclusion levels (Table 5). Breads supplemented with 10 % locust flour featured significantly higher (*p* < 0.05) crude fat, crude protein, fibre, ash and energy compared to diets with 0 % and 5 % inclusion levels. On the other hand, the control breads (0 % locust flour) recorded significantly higher carbohydrate and moisture contents. As a general trend, the proximate components (dry matter, protein, fat and ash) and energy proportionally increased with the increasing locust flour inclusion levels.

**Table 5:** Proximate components (g/100g dry matter) and gross energy content (kcal/100g) of wheat breads formulated with locust flour.

Proximate component	Locust flour inclusion level^a^			F_(2,6)_	p-value
	0 %	5 %	10 %		
Moisture content	31.73 ± 0.12^c^	30.07 ± 0.03^b^	27.10 ± 0.06^a^	874.76	0.001
Dry matter	68.27 ± 0.12^a^	69.93 ± 0.03^b^	72.90 ± 0.06^c^	874.76	0.001
Protein content	11.34 ± 0.12^a^	13.30 ± 0.15^b^	15.75 ± 0.44^c^	86.98	0.001
Fat content	2.32 ± 0.04^a^	3.71 ± 0.12^b^	4.96 ± 0.06^c^	266.56	0.001
Fibre content	1.35 ± 0.04^a^	1.23 ± 0.05^a^	1.79 ± 0.08^b^	22.22	0.01
Ash content	1.91 ± 0.02^a^	2.01 ± 0.03^b^	2.19 ± 0.02^c^	41.32	0.001
Carbohydrates content	49.38 ± 0.06^c^	47.38 ± 0.18^b^	45.50 ± 0.54^a^	34.02	0.001
Energy content	11.40 ± 0.00^a^	11.96 ± 0.03^b^	12.43 ± 0.17^c^	27.81	0.001

a Values are presented as mean with standard errors (±). Values in a row followed by different small letters (superscripts) within rows showed levels of significant difference at 5 %.

### 3.5 Amino acids profile of bread

Exactly fourteen amino acids (AAs) were identified from bread formulated with locust meal (Table 6). The dominant amino acids in the breads were phenylalanine and valine for essential AAs and glycine and proline for the non-essential AAs. All the amino acids except lysine, differed considerably (*p* < 0.05) following increasing wheat substitution levels. Breads enriched with locust flour expressed significantly higher levels of leucine, valine, isoleucine, threonine and histidine for the EAA and glycine, alanine, arginine, proline and tyrosine for the NEAA. The proportions of EAA and NEAA ranged 48.65–49.78 % and 50.22–51.35 %, respectively. The ratio of EAA/NEAA ranged between 0.95–0.99.

**Table 6:** Concentration (mg/g DM) of amino acids recorded from bread supplemented with locust flour.

	Proportions of locust flour					
Amino acid	0 %	5 %	10 %	F_(2,6)_	P value	WHO/FAO/UNU Amino acid requirements (mg/kg body weight/Day) (WHO et al., 2007)
**Essential amino acids (EAA)**§						
Phenylalanine	5.74 ± 0.51^a^	6.21 ± 0.27^ab^	6.84 ± 0.11^b^	7.83	0.05	25
Lysine	1.28 ± 0.03^a^	1.44 ± 0.38^a^	1.48 ± 0.21^a^	0.531	ns	30
Leucine	2.85 ± 0.20^a^	3.57 ± 0.09^b^	3.93 ± 0.08^c^	51.98	0.001	39
Valine	5.70 ± 0.29^a^	7.10 ± 0.32^b^	7.75 ± 0.48^b^	23.38	0.01	26
Methionine	0.44 ± 0.02^a^	0.52 ± 0.02^ab^	0.58 ± 0.06^b^	9.676	0.05	10.4
Isoleucine	1.16 ± 0.03^a^	1.41 ± 0.05^b^	1.56 ± 0.12^b^	19.57	0.01	20
Threonine	1.05 ± 0.07^a^	1.24 ± 0.09^b^	1.47 ± 0.06^c^	25.18	0.01	15
Histidine	1.41 ± 0.06^a^	2.11 ± 0.10^b^	2.17 ± 0.03^b^	122.8	0.001	10
**Non-essential amino acids (NEAA)**§						
Glycine	11.52 ± 0.73^a^	12.94 ± 0.09^b^	14.14 ± 0.37^c^	22.94	0.01	–
Alanine	0.00 ± 0.00^a^	1.03 ± 0.03^b^	1.22 ± 0.21^b^	85.27	0.001	–
Arginine	2.55 ± 0.10^a^	3.35 ± 0.10^b^	3.38 ± 0.20^b^	34.08	0.001	–
Glutamic acid	1.76 ± 0.10^a^	2.04 ± 0.09^ab^	2.09 ± 0.14^b^	7.739	0.05	–
Proline	3.85 ± 0.23^a^	4.90 ± 0.16^b^	5.07 ± 0.23^b^	29.25	0.001	–
Tyrosine	0.53 ± 0.01^a^	0.65 ± 0.03^b^	0.65 ± 0.07^b^	7.153	0.05	25
**∑EAA**	19.63	23.60	26.32			
**∑NEAA**	20.21	24.91	26.55			
**∑AA**	39.84	48.51	52.87			
**%EAA**	49.27	48.65	49.78			
**%NEAA**	50.73	51.35	50.22			
**EAA/NEAA**	0.97	0.95	0.99			

§ Values are expressed as averages with ± standard deviation (SD). Values in rows followed by different small letter superscripts within rows represents significant difference at 5 % level. WHO-World Health Organization; FAO-Food and Agriculture Organization; UNU-United Nations University.

### 3.6 Mineral content

Five macro-minerals and four trace minerals were found in the locust enriched breads and the control breads (Table 7). Sodium, iron and zinc were the most abundant minerals identified. The mineral levels (except for magnesium) significantly (*p* < 0.05) varied among the locust-based breads and their control counterparts. The 5 and 10 % locust flour enriched breads exhibited higher levels of calcium, phosphorous, sodium, iron and copper than the control bread.

**Table 7:** Mineral content (mg/100g DM) of breads formulated with different levels of locust flour.

	Locust flour inclusion levels					
Macro-minerals	0 %	5 %	10 %			FAO/WHO Recommended daily mineral intake (mg) (Lewis, 2019)
Calcium	0.02 ± 0.00^a^	0.04 ± 0.00^b^	0.05 ± 0.00^c^	36.96	0.001	1000
Phosphorous	0.12 ± 0.00^a^	0.13 ± 0.00^b^	0.14 ± 0.00^c^	23.55	0.01	700
Magnesium	0.02 ± 0.00^a^	0.03 ± 0.01^a^	0.03 ± 0.00^a^	0.91	ns	3
Sodium	173.23 ± 0.46^a^	205.80 ± 4.65^b^	298.77 ± 10.44^c^	97.24	0.001	–
Potassium	0.14 ± 0.00^a^	0.16 ± 0.00^a^	0.25 ± 0.02^b^	23.76	0.01	–
Trace minerals†						
Iron	11.34 ± 0.03^a^	12.77 ± 0.22^b^	15.21 ± 0.23^c^	112.69	0.001	14
Zinc	3.63 ± 0.07^a^	3.88 ± 0.08^a^	4.65 ± 0.12^b^	33.76	0.001	14
Manganese	0.63 ± 0.01^a^	0.75 ± 0.01^a^	1.25 ± 0.07^b^	67.02	0.001	3
Copper	0.27 ± 0.03^a^	0.37 ± 0.01^b^	0.61 ± 0.02^c^	72.75	0.001	0.06

† Values are presented as averages with ± standard error (SE). Means in rows followed with different small superscript letters indicates significant difference (*p* < 0.05).

### 3.7 Multivariate analysis of locust flour inclusion levels, the nutritional components and physical properties of the resultant breads

**Fig. 4:**
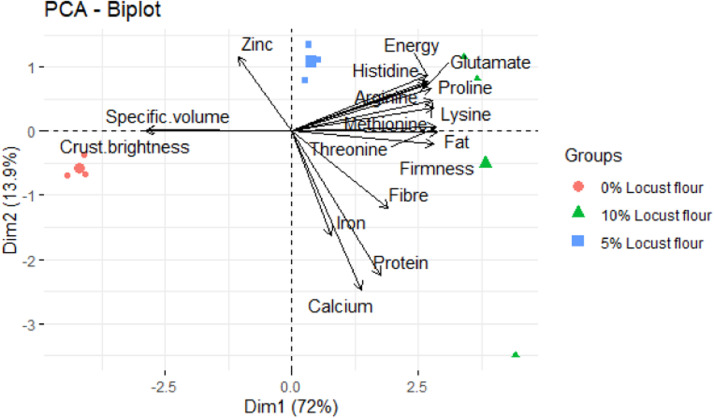
A PCA biplot presenting the relationship and patterns between the locust flour inclusion levels and the nutritional contents of the bread.

Fig. 4 illustrates multidimensional relationships among the nutritional components, physical properties and the locust flour inclusion levels. Amino acids (lysine, methionine, threonine, proline, glutamate, arginine and histidine), energy, protein, fibre, essential minerals (iron and calcium) and firmness were strongly associated with 10 % locust flour enriched bread. On the other hand, specific volume and crust brightness were strongly associated with the control bread. Specific volume negatively correlated with the bread firmness, amino acids and proximal components whereas crust brightness negatively correlated with reactive amino acids such as lysine, arginine and histidine that are often engaged in Maillard reactions.

### 3.8 Volatile traps from bread products

**Fig. 5:**
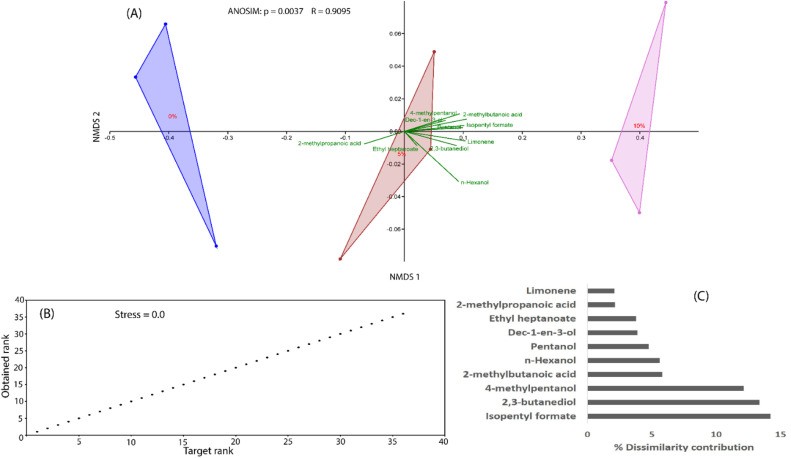
(A) Relationship of the different bread types based on specific volatiles emitted. (B) Shepard plot illustrating the great ordination of non-metric multidimensional- scaling plot analysis (stress value=0.01571). (C) Contribution of ten utmost important volatiles that differentiated the various bread types.

Fifty-three [53] volatile organic compounds (VOCs) were trapped from the control bread samples (0 % locust flour) and locust enriched bread samples (see supplementary Table 1). The compounds were classified into alcohols (15), aldehydes (11), heterocyclic compounds (9), hydrocarbons (5), esters (5), acids (3), ketone (3) and terpenes (2). Fig. 5A and Fig. 5B revealed that isopentyl formate, pentanol, 2,3-butanediol, 2-methylpropanoic acid, n-hexanol, 4-methyl-pentanol, 2-methyl-butanoic acid, dec‑1-en-3-ol, limonene and ethyl heptanoate were the key compounds that significantly contributed to the differentiation of the bread types (Table 8 & Fig. 5C).

**Table 8:** Principal volatile compounds recorded from the different bread types fortified with desert locust flour identified by non-metric multidimensional scaling plot.

tR	Compound	Compound class	Locust flour levels			F	DF	*p*-Value	Odour descriptors
			0 %	5 %	10 %				
4.64	Isopentyl formate	Ester	–	6.03 ± 1.05^a^	13.44 ± 1.77^b^	12.99	1,4	0.05	–
5.44	Pentanol	Alcohol	2.25 ± 0.16^a^	4.52 ± 0.81^ab^	6.60 ± 0.66^b^	12.6	2,6	0.01	Balsamic, fruity, fusel-like sweet
5.96	2,3-butanediol	Alcohol	9.90 ± 1.11^a^	12.52 ± 3.31^a^	22.87 ± 1.94^b^	8.85	2,6	0.05	Neutral smelling
6.02	2-methyl-propanoic acid	Acid	1.52 ± 0.20^a^	2.17 ± 0.20^a^	–	5.25	1,4	ns	Sweaty, butter, fatty, sour, rancid
8.25	n-Hexanol	Alcohol	7.57 ± 0.46^a^	8.51 ± 0.22^a^	13.28 ± 1.28^b^	14.73	2,6	0.01	Green grass, flowery, woody, mild, sweet
8.26	4-methylpentanol	Alcohol	–	–	13.29 ± 1.38	–	–	–	–
8.66	2-methylbutanoic acid	Acid	–	1.74 ± 0.39^a^	5.74 ± 0.38^b^	68.89	1,4	0.01	Cheese, rancid, sweaty
10.69	Dec-1-en-3-ol	Alcohol	1.36 ± 0.15^a^	3.87 ± 0.16^b^	–	131.72	1,4	0.001	–
11.63	Limonene	Cyclic Terpene	0.81 ± 0.08^a^	1.97 ± 0.11^b^	2.69 ± 0.21^c^	42.23	2,6	0.001	Citrus
12.86	Ethyl heptanoate	Ester	–	3.20 ± 0.30^b^	0.63 ± 0.03^a^	70.74	1,4	0.01	Grapes

Values are presented as mean ± standard error (SE). Means in rows followed with different small superscript letters indicates significant difference (*p* < 0.05). tR-retention time. Odour descriptors were adopted from Pico, Bernal and Gómez (2015).

## 4 Discussion

Great disparities in the nutritional contents of the experimental host plants were witnessed especially with regards to their protein contents. This is because legumes (*Phaseolus vulgaris*) and cereals (*Triticum aestivum* and *Sorghum bicolor*) have inter-species distinct roles in seed development. Beans prioritize protein storage for seed development and germination (Sathe, 1996) whereas wheat and sorghum prioritize accumulation of carbohydrate for energy (Rooney & Serna-Saldivar, 2003; Shewry, 2009). Several authors have affirmed that the nutritional composition of insects is a function of the chemical composition of their diet (Finke, 2013; Oonincx & van der Poel, 2011; Sangha et al., 2024). Therefore, differences spotted in the nutritional make-up of host plants may translate into variations in the chemical contents of locust they are fed on.

The crude protein level of 5th instar, immature adults and ovipositing females of the desert locust ranged between 40.31 and 55.81 % on dry matter (DM) basis which is in line with 15–81 % protein reported in Orthopteran species including locusts (Rumpold & Schlüter, 2013) and coincides with 50–65 % reported from *Locusta migratoria* (Mohamed, 2015; Oonincx et al., 2011; Osimani et al., 2017) and 46.3–76.0 % in adult desert locust (Egonyu et al., 2021). These protein levels surpass the levels that have been reported in conventional protein sources such as beef, veal, lamb and mutton (Williams, 2007). This is an evidence that desert locusts are endowed with a wealth of nutrients which can be tapped for counteracting malnutrition thereby qualifying them as befitting sustainable alternative protein source to conventional meat sources such as beef (Rumpold & Schlüter, 2013). Interestingly, desert locust fed on *Phaseolus vulgaris* expressed higher protein contents than those fed on the other food crops. In another study, locust species: *Cantantops melanostictus, Acanthacris ruficornis, Petamella prosternalis, Locusta migratoria, Cataloipus zulvensis, Chortoicetes terminifera* (Walker, 1870) and *Ornithacris cyanea* expressed higher feed intake, higher reproductivity, lower mortality and greater adult longevity when presented with *Phaseolus vulgaris* than when presented with combination of or single feeding plant species (Mphephu, 2023). Elsewhere, *S. gregaria* was reported to rapidly populate when solely fed on nitrogen rich *Heliotropium arbainesnse* (Fresen.) (Boraginaceae) while depicting low fecundity when fed on low nitrogen feed plant, *Panicum turgidum* (Forssk.) (Poceae) (van Huis et al., 2008). High protein levels in *Phaseolus vulgaris-*fed locusts maybe due to their high feed rate for *Phaseolus vulgaris* to build their body reserves than the other food crops. The high feed rate may have been occasioned by locust preference for high protein/nitrogen content (Mphephu, 2023) which *Phaseolus vulgaris* exhibited in higher quantities than in *Triticum aestivum* and *Sorghum bicolor* (Table 2). The present study revealed that the desert locust contain high amount of fat ranging between 4 and 22 % fat, which agrees with 13.0–32.3 % reported by Egonyu et al. (2021) and concords the average of 13 % fat content documented by Paul et al. (2017), on orthopteran insect species.

The levels of protein and fat in locusts observed in the current study compares with or supersedes that of conventional plant and animal origins, thereby affirming their potential as sustainable alternatives suitable for successful utilization by the linearly booming world populace (Yi et al., 2013; van Huis et al., 2013). Besides the protein and fat reported in the present study, additional evidence has shown that desert locusts are also rich in sterols, especially phytosterols. These phytosterols have been shown to impart cardio-friendliness to human health by suppressing plasma low density lipoproteins (Cheseto et al., 2015), modulating endothelial functionalities and antioxidant capacity (Marinangeli et al., 2006). Such pharmacological benefits, compounded with appealing sensory properties, rich nutritional profiles, and sustainable prospects of production as a food ingredient, have been the motivation underscoring the concerted interest towards the growing consumption of locust (van Huis et al., 2013; Mariod et al., 2017). To that effect, the narrative of considering desert locust as pests has been changed into their consideration as food evidenced by a spur-up of companies which creatively and purposely integrate the locust in floured form into modern products and meals (Clarkson et al., 2018). For instance, in New Zealand, the Food Standards Australia New Zealand (FSANZ) has categorized desert locusts as novel foods while exempting other three species, due to safety concerns. Several research accomplishments have demonstrated reasonably high levels of microbial hazards in fresh insects (Grabowski & Klein, 2017; Caparros-Megido et al., 2017). Therefore, to render locusts and other edible insect species fit for human consumption, several thermal and non-thermal processing steps have been proposed to downscale the safety risks associated with unprocessed insect species to recommendable levels (Vandeweyer et al., 2017; Caparros-Megido et al., 2017).

The negative association of bread specific volume with increasing locust flour ingredients echoed a phenomenon witnessed in breads formulated with cinereous cockroach, *Oncorhynchus tschawytscha* powder and mealworm powder (de Oliveira et al., 2017; Desai et al., 2018; Roncolini et al., 2019). Supplementation with protein rich locust flour may have disoriented the proportional balance of glutenin and gliadin protein values of wheat gluten as well as their covalent and hydrogen bond networks resulting into less viscoelastic doughs than in the control doughs (Bawa et al., 2020). Reduced viscoelasticity of doughs parallels rigidity of gas cells minimizing their tendency to expand, consequently reducing the exact volume of the bread products with increased inclusion levels of locust flour (de Oliveira et al., 2017). Conversely, the progressive increment in bread firmness with locust flour supplemental levels were consistent with the results on bread developed with 5 % mealworm flour (Roncolini et al., 2019) and breads fortified with cricket flour (Osimani et al., 2018). The increase in the bread firmness may be attributed to the excessive cross linking within the bread crumb matrix (Moore et al., 2006) occasioned by the increasing levels of incorporated locust flour. This phenomenon culminates into compacted bread crumps characterized by reducing specific volumes. The negative association between bread firmness and specific volume has been widely witnessed in breads by authors (Bresciani et al., 2022; Moore et al., 2006; Ronda et al., 2015). Generally, the reduction in crust and crumb brightness (L*) with increasing redness (a*) was initiated by the additive effects of locust flour to yield dark-brown bread (Fig. 3). The darkened colour of bread with increasing levels of locust flour can be attributed to intense non-enzymatic reactions (caramelization and Maillard reactions) engaging amine groups (amino acid and protein) and carbonyl groups (reducing sugars) as reactants (Desai et al., 2018; Roncolini et al., 2019). Temperature and cooking time has also been reported to influence colour change of crust and crumb of bread (González et al., 2019). A dark brown colour in bakery products is often regarded by consumers as nutritious and wholesome (Sandvik et al., 2018). The continuing increase in total colour difference (∆*E*) of both crust and crumb agrees with reports by Zielińska et al. (2021) on muffins fortified with *Tenebrio molitor* and *Gryllodes sigillatus* flours.

The moisture content (and corresponding dry matter content) observed in present study concorded the 23.33–31.24 % reported for whole wheat bread products enriched with cricket flour (Mafu et al., 2022) and 29.19–31.65 % reported on breads fortified with mealworm powder (Roncolini et al., 2019). The moisture contents however dropped with increasing locust flour supplementation, a phenomenon that may have been caused by the presence of less reactive hydrophilic groups or low water-binding capacity proteins and amino acids dominating the locust flour (González et al., 2019). The protein levels spectacularly escalated with additive levels of the locust flour inclusion resulting into 10 % locust flour incorporated breads with protein contents surpassing the minimum 15 % level recommended for insect-based food products by Kenya Bureau of Standards (KEBS) (KEBS, 2020). Proteins is the main macromolecular component of desert locusts (Table 3) and therefore, blending of the wheat flours with increasing levels of such substrates may have been reflected in the bread products. A similar trend was evidenced when wheat breads were formulated with varying levels of *Locusta migratoria* flours (Althwab et al., 2021), cinereous cockroach flour (de Oliveira et al., 2017), *Acheta domesticus* powder (Kowalczewski et al., 2021; Mafu et al., 2022) and *Tenebrio molitor* powder (Roncolini et al., 2019). It is therefore an irrefutable fact that insect ingredients possess the potential of enhancing the nutritional contents of commonly consumed products of plant origin. Since current dietary frontiers are in favour of high protein-low energy food sources (Pauter et al., 2018), the utilization of desert locust in this study could transform the notion of locusts’ consideration as threat to food security to a contributor to food security. Based on the 50 g/day Recommended Dietary Allowance (RDA) prescribed by FAO and WHO (Lewis, 2019), consumption of 100 g of the control breads could contribute to 22.6 % of the protein RDA whereas the locust-wheat flour breads could contribute to 26.6–31.5 % of the protein RDA. The locust fortified breads compared to the reference breads in the current study, can therefore serve as nutritionally relevant products that significantly contribute to the Recommended Dietary Allowance (RDA) for protein (WHO et al., 2007) hence ideal for advancing human nutrition. However, there is need for biodigestibility and antinutritional studies to determine the nutritional quality and value of the locust proteins. Fascinatingly, Melo et al. (2011) reported the biodigestibility of *Schistocerca gregaria* protein to be 89–92 % which is comparable to the digestibility of milk casein. Such a high digestibility may be associated with the reportedly low levels of antinutrients such as chitin, tannins, oxalates and phytates in locust (Bukkens, 1997; Fombong et al., 2017; Kinyuru et al., 2013; Melo et al., 2011) which are known to negatively correlate with biodigestibility of nutrients.

The linear increment in the fat and ash contents with increasing wheat-locust flour substitution levels corroborated the findings documented by Althwab et al. (2021) who attributed it to higher proximal components of the fortified breads with higher levels of *Locusta migratoria* flour. This report can apparently be verified in Table 2, where the desert locust flours displayed significantly higher fat and ash than the wheat flour. Ash contents collectively represents all available minerals in a food whereas fats are sources of essential fatty acids instrumental to human health (Althwab et al., 2021). This suggests that desert locusts utilized in this study are endowed with nutritionally valuable compounds. Despite the other proximate components’ positive correlation with the locust flour enrichment levels, the carbohydrate values depicted a negative association. This is ascribed to the substitution of high carbohydrate wheat powder with high protein locust powder, culminating into a wheat-carbohydrate dilution effect, due to mass balance. The low carbohydrate content of desert locust has previously been reported (Zielińska et al., 2018). The bread products can therefore be ideal diets for obese and diabetic patients. A similar scenario was witnessed when breads were formulated with cricket flour (Mafu et al., 2022). Despite the declining carbohydrate values recorded for bread products with increasing inclusion levels, the gross energy of the supplemented breads was observed to increase significantly. This may be attributed to the increasing levels of fat and protein which significantly correlates with calorie levels of a food (Kinyuru, 2020).

Amino acids are subunits of proteins whose spectra and concentrations denote the protein quality of a food. Insects have been poised as valuable sources of quality amino acids suitable for counteracting malnutrition (van Huis et al., 2021). The locust flour adopted in this study for revamping the nutritional content of breads relative to the reference breads are not an exception, despite their limited consideration as food. Fortification of bread with varying locust flour levels altered all the amino acids contents except lysine, despite its previous considerable detection (35.1 mg/g) from commercially reared *Schistocerca gregaria* (Zielińska et al., 2015). The lack of statistical variability of lysine may be attributed to their reactive engagement with sugars during Maillard reactions yielding dark brown bread crust and aroma compounds in the locust-flour supplemented breads (Desai et al., 2018; Ogur, 2014) compared to the control breads. The dark-brown crust in breads and other bakery products are appealing properties to consumers as it is associated to with wholesomeness of the product. Therefore, since wheat protein (gluten) principally comprises of proline and glutamine only, lysine is always limiting amino acid in wheat flours and some of its derived products. Unfortunately, correcting this deficiency was not achieved in this study, hence, future studies should endeavor to optimize baking conditions to preserve the biological value of such a crucial amino acid. The abundant display of the indispensable amino acids (phenylalanine, leucine and valine) and dispensable amino acids (glycine, proline and arginine), mirrored their dominant levels previously reported in commercially reared *Schistocerca gregaria* (Zielińska et al., 2015). Overall, the current study successfully enhanced the amino acids levels in the breads enriched with locust flour relative to the standard bread. Similar tendency manifested when bread was developed with mealworm powder (Roncolini et al., 2019) and *Locusta migratoria* powder (Althwab et al., 2021). Interestingly, consuming 100 g of the breads supplemented with the locust flours could potentially surpass the RDA of the essential amino acids (Table 6) as stipulated by WHO and FAO (WHO et al., 2007). However, it is noteworthy that this is also subject to the body weight of the consumer.

Standard bread were characterized with lower mineral contents since they were formulated with unsubstituted refined wheat flour which has previously been demonstrated to exhibit lower ash and minerals probably as results of the removal of bran and germ during processing (i.e., milling) (Ali et al., 2020). The mineral levels (except magnesium) in the locust flour enriched breads, particularly those incorporated with 10 % locust flours were markedly improved possibly due to the additive effect from the locust flour. Similar occurrence has been witnessed in other studies that attempted to integrate insect ingredients into bakery products (Althwab et al., 2021; Ayensu et al., 2019; Bawa et al., 2020). *Schistocerca gregaria* has been reported to furnish appreciable levels of potassium, phosphorus, sodium, calcium, zinc and iron as the predominant minerals that can significantly play a crucial role in human nutrition and health (Kinyuru, 2020). The implication of the locust flour fortified breads with high levels of calcium, iron and zinc relative to the control breads justifies the need for the utilization of wild collected desert locust to indirectly alleviate micronutrient deficiencies that is ubiquitous among children and pregnant mothers. Enhancement of micronutrients in food products through enrichment is reminiscent of other widely adopted nutritional enhancement techniques such as biofortification that have successfully elevated micronutrient elements in micronutrient deficient food crops (Malézieux et al., 2024). In the present study, the insect-fortified breads could contribute 91.2–108.6 % of the recommended nutrient intake for iron compared to the reference bread's 81.0 % contribution. On the other hand, the insect-based breads could contribute to 27.7–33.2 % of the dietary zinc requirements according to the advocacies by WHO and FAO (Lewis, 2019). Iron, zinc and calcium are essential minerals pivotal to basic metabolic body operations (Ayensu et al., 2019).

Volatile compounds dictates the flavour, aroma and taste of a food which in turn influences consumer's receptiveness (Ochieng et al., 2023a). The gustative and olfactory impressions experienced during ingestion of bread are dependent on flavour and the physical traits (volume, texture and colour) that are consumer indicators for bread quality (Pico et al., 2015). Intriguingly, consumers often discriminate a product principally based on aroma which is the first attribute perceive by their olfactory senses. The classes of volatile organic compounds detected are in line with the various aroma compounds previously reported to emanate from bread crumbs and crust (Pico et al., 2015). The compounds that contributed to the dissimilarities of the three bread products as visualized by the non-metric multidimensional scaling were mostly associated with 5 % and 10 % locust flour enriched products, signifying the relevance of the locust flour in conferring flavouring agents to such products. Some of these compounds, classified as alcohols and esters may have accumulated as result of baking-mediated lip-oxygenation of unsaturated fatty acids (Pico et al., 2015) since the enriched breads were fortified with locust flour reportedly rich in oleic, linoleic and linolenic acids (Kinyuru, 2020). Nissen et al. (2020) equally detected a set of new and unique volatile compounds: 1-hexanol, nonanoic acid, 2E,4E-nonadienal, 3-octen-2-one, and 1-heptanol, in breads enriched with cricket flour. Most aldehyde compounds and some pyrazines detected (Table S1) only in or significantly enhanced in the locust flour enriched breads may be hypothesized to be generated from Strecker degradation, a process involving the reaction of dehydroreductones with amino acids (higher levels in the enriched breads than control) to yield aldehydes with structures mimicking the reactive amino acid as well as melanoidines (brown pigments) resulting from Maillard reactions (Picoet al., 2015). The dominant class of alcoholic compounds was expected since most volatiles associated with bread are derived from anaerobic fermentation of bread doughs using yeast and lactic acid bacteria during leavening. The yeasts may also assimilate the amino acids in dough substrate and excrete them as alcohols, acids and aldehydes (Pico et al., 2015).

## 5 Conclusion

**Fig. 6:**
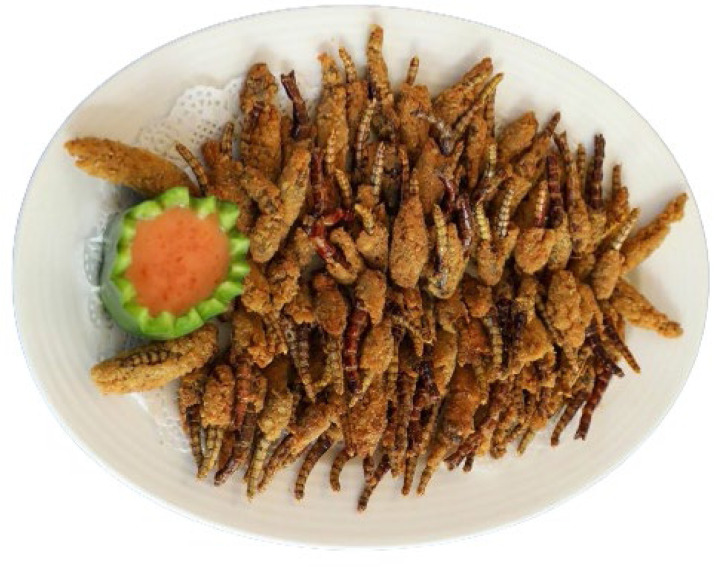
“Plaque to Plate”: ICIPE turns to innovative technologies to transform the devastating locust swarms into amazing superfood resource.

The fact that substituting wheat flour with increasing inclusion levels of desert locust meals linearly enhanced the most essential nutrients in breads reinforce the global advocacy for sustainable diets for improved health and nutrition. This suggests that desert locust meal could be considered as novel functional ingredients ideal for food fortification conferring proteins, essential amino acids and minerals to leavened breads with unbalanced nutrient profiles. The locust ingredients are also rich in valuable compounds that are essential to the engineering of heterocyclic compounds, aldehydes, ketones, esters and alcohols during leavening and baking of bread to yield pleasant aroma compounds that are pleasurable to consumers. The additive levels of locust flour also impart darker colours to bread crumbs which are attractive to consumers who can benefit from the nutritional valuables of the locust breads. This study paves way shelf-life determinations on formulated breads and sensory evaluation of the developed products to gauge consumer preferences that may inform the necessary modifications of future locust-based products to align with the gastronomic norms of target consumers. Desert locust can therefore be valorized as a food source through biological control (hand picking) rather than disastrous swarms frequently sprayed with massive volumes of pesticides in outbreak regions of sub-Saharan Africa. This study emulates Israel's “plaque on a plate” strategies (Fig. 6) by providing opportunities for entrepreneurs to transform the terror presented by locusts into a benefit for humankind through captive breeding of the dreaded insects to market a full range of consumer products. Locusts could be an amazing solution to the global search for a more sustainable and healthier source of Biblical Protein lines. In this regard, scientists from ICIPE are investigating how the destructive pests that cause widespread damage to crops could be turned into a form of sustenance for the estimated 1 billion people across Africa and Asia who suffers from the lack of protein in their diets.

## Funding sources

The research efforts were financially supported by the National Postcode Lottery [B(ea)T The Locust: 200952], Global Affairs Canada (BRAINS project: P011585), Novo Nordisk Foundation (RefIPro: NNF22SA0078466), Bill and Melinda Gates Foundation (INV-032416), The Australian Centre for International Agricultural Research (ProteinAfrica: LS/2020/154), the Rockefeller Foundation (2021 FOD 030), the Curt Bergfors Foundation Food Planet Prize Award, European Commission (NESTLER - Project: 101060762 - HORIZON-CL6-2021-FARM2FORK-01 and INNOECOFOOD project: 101136739), Norwegian Agency for Development Cooperation, the Section for Research, Innovation, and Higher Education [RAF–3058 KEN–18/0005], the Australian Centre for International Agricultural Research; the Norwegian Agency for Development Cooperation (NORAD); the Swedish International Development Cooperation Agency (SIDA); the German Federal Ministry for Economic Cooperation and Development (BMZ); the Swiss Agency for Development and Cooperation (SDC) and the Government of the Republic of Kenya. The funders had no role in study design, data collection and analysis, decision to publish, or preparation of the manuscript. The views expressed herein do not necessarily reflect the official opinion of the donors.

## Ethical statement - studies in humans and animals

According to strict adherence to all the provisions provided by the Institutional Animal Care and Use Committee (IACUC) of Kenya Agricultural and Livestock Research Organization (KALRO)-Veterinary Science Research Institute (VSRI) [KALRO-VSRI/IACUC028/16032022], Muguga North, this research work was approved for implementation. Furthermore, this study was assessed and ratified by the National Council for Science Technology and Innovation in Kenya (NACOSTI/P/21/8303) and the University of Nairobi, Nairobi, Kenya, which provides permission to undertake sample collection from the field, processing, identification and experimental research in the laboratory.

## CRediT authorship contribution statement

**Chrysantus M. Tanga:** Writing – review & editing, Writing – original draft, Visualization, Validation, Supervision, Software, Resources, Project administration, Methodology, Investigation, Funding acquisition, Formal analysis, Data curation, Conceptualization. **Antonny M. Nzomo:** Writing – review & editing, Visualization, Validation, Methodology, Investigation, Formal analysis, Data curation, Conceptualization. **Paul N. Ndegwa:** Writing – review & editing, Visualization, Validation, Supervision, Methodology, Conceptualization. **Sunday Ekesi:** Writing – review & editing, Visualization, Validation, Project administration. **Fathiya M. Khamis:** Writing – review & editing, Visualization, Validation, Conceptualization. **Komivi S. Akutse:** Writing – review & editing, Visualization, Validation, Conceptualization. **George Ong'amo:** Writing – review & editing, Visualization, Validation, Supervision, Methodology, Conceptualization. **Brian O. Ochieng:** Writing – review & editing, Visualization, Validation, Formal analysis, Data curation. **Margaret Kababu:** Writing – review & editing, Visualization, Validation, Formal analysis, Data curation. **Dennis Beesigamukama:** Writing – review & editing, Visualization, Validation, Formal analysis, Data curation. **Shaphan Y. Chia:** Writing – review & editing, Visualization, Validation, Formal analysis, Data curation. **J․Ghemoh Changeh:** Writing – review & editing, Visualization, Validation, Investigation, Conceptualization. **Sevgan Subramanian:** Writing – review & editing, Visualization, Validation, Project administration. **Thomas Dubois:** Writing – review & editing, Visualization, Validation, Project administration. **Segenet Kelemu:** Writing – review & editing, Visualization, Validation, Project administration, Funding acquisition, Conceptualization.

## Declaration of competing interest

The authors declare that they have no known competing financial interests or personal relationships that could have appeared to influence the work reported in this paper.

## Data Availability

Data will be made available on request.

## Appendix Supplementary materials

Image, application 1
