# Supplementary material for: Desert locust (Schistocerca gregaria) flour as an emerging functional ingredient for baking flavorful and nutritious whole wheat bread
Source: Appl Food Res. 2025 Jun;5(1):None. doi: 10.1016/j.afres.2025.100802 (PMC12167175; doi:10.1016/j.afres.2025.100802)
Supplement: Supplementary file 1 [file mmc1.docx]

# **Supplementary information**

**Table S1:** Volatile organic compounds (µg/g/h) of the control and locust flour enriched breads analysed by GC-MS.

| **rT** | **Compound** | **Compound class** | **Locust flour incorporation levels** | | | **F** | **DF** | **P-Value** | **Odour descriptors** |
| --- | --- | --- | --- | --- | --- | --- | --- | --- | --- |
|  |  |  | **0%** | **5%** | **10%** |  |  |  |  |
| 3.34 | 1-methoxy-2-methyl-1-propene | Hydrocarbon | - | 0.06 ± 0.00 | - | - | - | - |  |
| 3.53 | Pentanal | Aldehyde | - | 0.17 ± 0.01^a^ | 0.48 ± 0.04^b^ | 61.48 | 1,4 | 0.01 | Strong acrid, pungent |
| 4.64 | Isopentyl formate | Ester | - | 6.03 ± 1.05^a^ | 13.44 ± 1.77^b^ | 12.99 | 1,4 | 0.05 | - |
| 4.68 | 2-methyl-1-butanol | Alcohol | - | 0.16 ± 0.01 | - | - | - | - | Malty |
| 5.03 | 2E-penten-1-al | Aldehyde | - | 0.08 ± 0.00 | - | - | - | - |  |
| 5.44 | Pentanol | Alcohol | 2.25 ± 0.16^a^ | 4.52 ± 0.81^ab^ | 6.60 ± 0.66^b^ | 12.6 | 2,6 | 0.01 |  |
| 5.71 | 3-Penten-2-ol | Alcohol | - | 0.36 ± 0.02 | - | - | - | - |  |
| 5.96 | 2,3-butanediol | Alcohol | 9.90 ± 1.11^a^ | 9.18 ± 0.54^a^ | 22.87 ± 1.94^b^ | 33.71 | 2,6 | 0.001 | Neutral smelling |
| 6.02 | 2-methyl-propanoic acid | Acid | 1.52 ± 0.20^a^ | 2.17 ± 0.20^a^ | - | 5.25 | 1,4 | ns | Sweaty, butter, fatty, sour, rancid |
| 6.99 | Methylpyrazine | Heterocyclic compound | 0.17 ± 0.01^a^ | 0.53 ± 0.22^a^ | 0.29 ± 0.01^a^ | 2.02 | 2,6 | ns |  |
| 7.27 | Furfural | Heterocyclic compound | 0.36 ± 0.02^b^ | 0.80 ± 0.05^c^ | 0.15 ± 0.01^a^ | 98.16 | 2,6 | 0.001 | Almond, bread-like, soil, burnt roasted, sweet, toasted |
| 7.28 | 3-Furaldehyde | Aldehyde | - | 0.70 ± 0.03 | - | - | - | - |  |
| 7.34 | 2-methyl-1-pentanol | Alcohol | 0.29 ± 0.02^c^ | 0.17 ± 0.01^b^ | 0.10 ± 0.00^a^ | 48.09 | 2,6 | 0.001 |  |
| 7.51 | 3-ethoxy-1-propanol | Alcohol | 0.20 ± 0.01^a^ | 0.29 ± 0.02^b^ | 0.39 ± 0.03^c^ | 22.91 | 2,6 | 0.01 | Fruit |
| 7.90 | 2-Furanmethanol | Alcohol | 2.33 ± 0.20^b^ | 1.54 ± 0.09^a^ | 2.15 ± 0.20^ab^ | 5.93 | 2,6 | 0.05 |  |
| 8.16 | o-Xylene | aromatic hydrocarbon | 0.21 ± 0.01 | - | - | - | - | - |  |
| 8.25 | n-Hexanol | Alcohol | 7.57 ± 0.46^a^ | 8.51 ± 0.22^a^ | 13.28 ± 1.28^b^ | 14.73 | 2,6 | 0.01 | Green grass, flowery, woody, mild, sweet |
| 8.26 | 4-methyl-pentanol | Alcohol | - | - | 13.29 ± 1.38 | - | - | - |  |
| 8.66 | 2-methyl-butanoic acid | Acid | - | 1.74 ± 0.39^a^ | 5.74 ± 0.38^b^ | 68.89 | 1,4 | 0.01 | Cheese, rancid, sweaty |
| 9.01 | Heptanal | Aldehyde | 0.56 ± 0.04^a^ | 0.64 ± 0.04^a^ | 1.14 ± 0.11^b^ | 19.21 | 2,6 | 0.01 | Fatty, rancid, citrus, malty |
| 9.10 | 2-butoxy-ethanol | Alcohol | 1.65 ± 0.05^b^ | 1.13 ± 0.16^a^ | 1.60 ± 0.09^ab^ | 6.79 | 2,6 | 0.05 |  |
| 9.19 | 2,5-dimethylpyrazine | Heterocyclic compounds | 0.57 ± 0.10^a^ | 0.83 ± 0.15^a^ | 1.93 ± 0.13^b^ | 31.48 | 2,6 | 0.001 | Crust-like, popcorn |
| 9.25 | 4-hydroxybutanoic acid | Acid | 0.64 ± 0.11^a^ | 0.88 ± 0.03^a^ | 1.46 ± 0.06^b^ | 29.79 | 2,6 | 0.001 |  |
| 9.38 | 2,3-dimethylpyrazine | Heterocyclic compounds | 0.62 ± 0.09^a^ | 0.43 ± 0.02^a^ | 1.59 ± 0.10^b^ | 65.44 | 2,6 | 0.001 | Popcorn, roasted |
| 10.01 | 1-methyl-2-propyl-cyclohexane | Hydrocarbon | 0.40 ± 0.02^a^ | 0.41 ± 0.03^a^ | - | 0.52 | 1,4 | ns |  |
| 10.16 | Dihydro-5-methyl-2(3H)-furanone | Heterocyclic compounds | - | 0.18 ± 0.01^a^ | 0.21 ± 0.01^a^ | 5.4 | 1,4 | ns | - |
| 10.22 | 2E-heptenal | Aldehyde | 0.62 ± 0.04^a^ | 1.09 ± 0.15^a^ | 2.22 ± 0.11^b^ | 55.91 | 2,6 | 0.001 | Green, fatty |
| 10.27 | Benzaldehyde | Aldehyde | 1.52 ± 0.090^a^ | 1.94 ± 0.13^ab^ | 2.22 ± 0.11^b^ | 9.9 | 2,6 | 0.05 | Almond, caramel |
| 10.52 | n-Heptanol | Alcohol | 0.65 ± 0.04^a^ | 0.72 ± 0.09^a^ | 0.54 ± 0.06^a^ | 2.32 | 2,6 | ns | Green |
| 10.69 | Dec-1-en-3-ol | Alcohol | 1.36 ± 0.15^a^ | 3.87 ± 0.16^b^ | - | 131.72 | 1,4 | 0.001 | - |
| 10.79 | 2,3-Octanedione | Ketone | - | 0.41 ± 0.03^a^ | 0.85 ± 0.02^b^ | 210.91 | 1,4 | 0.001 | - |
| 10.86 | 6-methyl-5-hepten-2-one | Ketone | 0.35 ± 0.02^a^ | 0.54 ± 0.02^b^ | 0.72 ± 0.04^c^ | 36.19 | 2,6 | 0.001 | Herbaceous, green |
| 10.93 | 2-Pentylfuran | Heterocyclic compound | 0.57 ± 0.04^a^ | 0.70 ± 0.06^ab^ | 0.83 ± 0.01^b^ | 8.83 | 2,6 | 0.05 | Butter, green bean, floral, fruity, mushroom, raw nuts |
| 11.10 | Ethyl hexanoate | Ester | - | 0.64 ± 0.02^a^ | 1.57 ± 0.06^b^ | 228.64 | 1,4 | 0.001 | Applepeel, fruity |
| 11.15 | n-Octanal | Aldehyde | 0.78 ± 0.11^a^ | 1.23 ± 0.13^b^ | 1.62 ± 0.06^b^ | 16.62 | 2,6 | 0.01 | Citrus, flowery |
| 11.40 | 2-ethenyl-6-methyl-pyrazine | Heterocyclic compounds | - | 0.91 ± 0.08^b^ | 0.16 ± 0.01^a^ | 85.14 | 1,4 | 0.001 | Nutty |
| 11.44 | 2-acetyl-thiazole | Heterocyclic compounds | - | 0.32 ± 0.02^a^ | 0.51 ± 0.02^b^ | 49.31 | 1,4 | 0.01 | Roasty |
| 11.45 | 4-Cyanocyclohexene | Hydrocarbon | 0.19 ± 0.02 | - | - | - | - | - | - |
| 11.63 | Limonene | Terpene | 0.81 ± 0.08^a^ | 1.97 ± 0.11^b^ | 2.69 ± 0.21^c^ | 42.23 | 2,6 | 0.001 | Citrus |
| 11.69 | 3-ethyl-2-methyl-1,3-hexadiene | Hydrocarbon | - | 0.57 ± 0.11^a^ | 0.72 ± 0.02^a^ | 1.97 | 1,4 | ns | - |
| 11.733 | Benzyl alcohol | Alcohol | - | - | 0.37 ± 0.02 | - | - | - | - |
| 11.92 | Benzeneacetaldehyde | Aldehyde | 0.68 ± 0.05^a^ | 1.60 ± 0.19^b^ | 2.41 ± 0.17^c^ | 35.68 | 2,6 | 0.001 |  |
| 12.17 | 2E-Octen-1-al | Aldehyde | - | - | 0.81 ± 0.01 | - | - | - | Fatty, nutty, roasted |
| 12.39 | n-Undecanol | Alcohol | - | - | 0.74 ± 0.01 | - | - | - | Fruity type |
| 12.58 | 3-ethyl-2,5-dimethyl-pyrazine | Heterocyclic compounds | 0.57 ± 0.39^a^ | 0.47 ± 0.03^a^ | 0.68 ± 0.07^a^ | 0.21 | 2,6 | ns | Baked potato-like, earthy |
| 12.76 | 2-Nonanone | Ketone | - | 0.16 ± 0.01 | - | - | - | - | Fruity |
| 12.86 | Ethyl heptanoate | Ester | - | 3.20 ± 0.30^b^ | 0.63 ± 0.03^a^ | 70.74 | 1,4 | 0.01 | Grape |
| 12.96 | n-Nonanal | Aldehyde | 1.88 ± 0.21^a^ | 2.73 ± 0.24^ab^ | 3.25 ± 0.14^b^ | 12.06 | 2,6 | 0.01 | Citrus, soapy |
| 13.13 | Phenylethyl alcohol | Alcohol | 1.59 ± 0.10^a^ | 2.62 ± 0.40^ab^ | 3.18 ± 0.13^b^ | 10.26 | 2,6 | 0.05 | Rose-honey-like, wilted rose |
| 14.32 | Ethyl-(4E)-octenoate | Ester | - | - | 1.58 ± 1.13 | - | - | - | - |
| 14.44 | Ethyl octanoate | Ester | 0.36 ± 0.02^a^ | 0.44 ± 0.02^a^ | 1.94 ± 0.22^b^ | 50.36 | 2,6 | 0.001 | Sweet, soap, fresh, fruity |
| 14.58 | n-Decanal | Aldehyde | - | 0.57 ± 0.06^a^ | 0.82 ± 0.01^b^ | 17.74 | 1,4 | 0.05 | Citrus |
| 17.66 | alpha-Cedrene | Terpene | 0.51 ± 0.02^a^ | 0.65 ± 0.16^a^ | - | 0.79 | 1,4 | ns | - |
| Odour descriptors were adopted from (Pico et al., 2015) | | | | | | | | | |
